# Turning Agricultural Waste Into Enzymatic Treasure: Bromelain Stability in Pineapple Crown and Peel Waste From Subang District, Indonesia

**DOI:** 10.1155/bri/7470038

**Published:** 2026-02-27

**Authors:** Nyi Mekar Saptarini, Driyanti Rahayu, Danni Ramdhani, Delphine Wirawan, Virgiana Keisha Rajabi Johni Sudirman

**Affiliations:** ^1^ Department of Pharmaceutical Analysis and Medicinal Chemistry, Faculty of Pharmacy, Universitas Padjadjaran, Sumedang, 45363, West Java, Indonesia, unpad.ac.id

**Keywords:** bromelain, pineapple crown, pineapple peel, protease activity, storage time, total protein content

## Abstract

Bromelain is one of the protease enzymes found in all parts of pineapple (*Ananas comosus* (L.) Merr.), including the crown and peel. This enzyme has been widely used in various fields of life, including the food industry, health, pharmaceuticals, and cosmetics. However, pineapple processing often focuses on the flesh of the fruit, leaving behind substantial agricultural waste, such as crown and peel waste. The waste is often collected and stored before being used, causing the bromelain enzyme to decrease or even dissipate. Therefore, this study aims to determine the effect of time and the condition of storage of pineapple crown and peel waste on total protein content and protease activity. The extracted bromelain was precipitated with ethanol and then dried, and total protein content and protease activity were determined. The results showed that pineapple crown and peel waste can be stored for 7 days at 29 ± 1°C and humidity of 70 ± 2% and 20 days at 4 ± 0.5°C and humidity of 40 ± 2%, respectively. The total protein content and protease activity were 169.94 ± 2.68 μg/mL and 46.35 ± 0.69 IU/mg for crown bromelain, while those for peel bromelain were 229.75 ± 15.61 μg/mL and 29.10 ± 1.98 IU/mg, respectively. In conclusion, pineapple crown waste has the potential to be developed as a source of bromelain.

## 1. Introduction

Indonesia is one of the five largest pineapple‐producing countries in the world [1]. In 2023, Indonesia produced 3,156,576 tons of pineapple [2], with 1,713,324 quintals produced in West Java [2]. The largest pineapple‐producing area in West Java is Subang, with a total production of 1,674,136 quintals [2]. Pineapple (*Ananas comosus* (L.) Merr.) is rich in flavonoids, tannins, phenolic acids, lignin, carotenoids, vitamin C, and bromelain [3]. The derivative products include jam, chips, syrup, and dodol. However, pineapple processing generates a substantial amount of waste, defined as the residual material from a production process that has no economic value [4]. Pineapple waste reaches approximately 60% (w/w) of the plant weight, including 56% peel, 17% crown, 15% bud, 7% stem, and 5% pulp [5]. As pineapple processing increases, waste production also rises [1]. Pineapple peel and crown waste are currently only used as animal feed and compost [6], despite containing flavonoids, saponins, tannins, and the enzyme bromelain, which can provide benefits [7].

Unprocessed waste becomes an environmental problem; hence, pineapple crown and peel waste are being used as an economically valuable raw material through the application of the circular economy concept. The circular economy is an economic model that aims to minimize the negative impacts of human activities by applying the principles of reduce, reuse, and recycle to maintain products, components, and materials in value and utility for a long period. This concept focuses on efforts to minimize environmental impacts and create a more sustainable system. The application of the circular economy includes waste utilization, the use of environmentally friendly packaging, energy conservation, the use of renewable energy, and product design that considers sustainability [8]. Pineapple waste is used by extracting the bromelain contained within.

Bromelain is a protease enzyme that breaks down proteins into amino acids. It is used in various industries, such as pharmaceuticals, food, textiles, and cosmetics [6]. Bromelain is known to have pharmacological activities as an anticancer, fibrinolytic, antimicrobial, and anti‐inflammatory agent [9]. Bromelain is generally recognized as safe (GRAS) according to the LiverTox database and is widely used in herbal and dietary supplements. Bromelain is also generally well‐tolerated in humans when taken orally or applied topically. Evaluations of liver safety have not identified clinically significant hepatotoxicity associated with oral or topical uses [10]. In the food industry, this enzyme is used as a meat tenderizer, beer clarifier, and to inhibit the fruit oxidation process. In the textile industry, bromelain can remove scale and dirt from wool and silk fibers [11]. The cosmetics industry uses the enzyme to treat various skin problems, such as acne and dry skin [12]. When using bromelain from pineapple crown and peel waste, it is important to monitor the stability, which in turn affects the activity. Generally, waste is processed after being collected for a certain period, allowing changes in the content. Furthermore, waste storage conditions also need to be considered to maintain the quality of bromelain. Peeled pineapple stored at 20°C showed rotting on the 4th day [13]. Conversely, storage at lower temperatures may slow the rotting process. Low temperatures potentially inhibit the growth of microorganisms [14]. This is supported by Thu et al., who showed that pineapple stored at 4°C experienced mold growth on the 14th day [13]. Fruit rot also affects the activity of the enzymes within. Changes in enzyme activity were observed by Ridzuan et al., who showed that protease enzyme activity in pineapple waste juice extract increased until the 6th day, then began to decline until the 10th day. These results show that the activity and quantity of bromelain enzymes in pineapple crown and peel waste have the potential to change during the collection period [15]. Therefore, this study aims to determine the effect of waste storage time on the total protein content and protease activity of bromelain to optimize the use of pineapple crown and peel waste.

## 2. Materials and Methods

### 2.1. Materials

Eight‐month‐old pineapples were collected from Subang District, West Java, Indonesia, and were identified in the Biosystematics and Molecular Laboratory, Department of Biology, Universitas Padjadjaran, with No. 451/LBM/IT/X/2024. Bromelain standard was obtained from pineapple stem (≥ 3 IU/mg protein), while bovine serum albumin (BSA), casein, tyrosine, trichloroacetate (TCA), Bradford reagent, hydrochloric acid, and sodium hydroxide of analytical grade were purchased from Sigma Aldrich.

### 2.2. Waste Preparation

The fruit was cleaned of soil and dust, then the crown and peel were separated and weighed. The crowns were stored at 29 ± 1°C and a humidity of 70 ± 2%, while peels were stored at 4 ± 0.5°C and a humidity of 40 ± 2%, respectively, with consideration of water content in the waste [15]. Storage was carried out until microbial growth was observed.

### 2.3. Bromelain Extraction

A total of 200 g of peels and 350 g of pineapple crowns were chopped and ground with distilled water at a ratio of 1:1 (peel) and 1:2 (crown). The mixture was filtered, and 96% ethanol (1:4) was added to the filtrate, then stored for 8 h at 4°C. The solution was centrifuged at 10,000 rpm for 15 min, and the precipitate was dried at 30°C [16]. Bromelain extraction was carried out until microbial growth was observed, namely on Days 0, 1, 3, 5, and 7 for the crown and Days 0, 4, 8, 12, 16, and 20 for the peel [15].

### 2.4. Determination of Total Protein Content

A total of 50 μL of BSA as the standard (25, 50, 100, 150, 300, and 400 μg/mL), extracted bromelain, and blank were each reacted with 200 μL of Bradford reagent for 5 min at room temperature. The absorbance was read at 595 nm [17] and then used to create the calibration curve. Total protein content was determined for all extracted bromelain according to storage time.

### 2.5. Determination of Protease Activity

The absorbance of tyrosine (25, 50, 100, 200, 300, and 400 μg/mL) was used to create a product standard curve. Blank, standard bromelain (0.5 mg/mL), and extracted bromelain (0.5 mg/mL) were each added to the casein solution (0.5 mg/mL), then incubated for 30 min. TCA solution was added, incubated in a 90°C water bath for 5 min, and cooled to room temperature. Absorbance was measured at 275 nm [18], and protease activity was calculated using formula (1). Protease activity was determined on all extracted bromelain according to storage time.

(1) Protease activity IU/mL=μg/mL tyrosine produced×dilution factorBromelain volume×time.

### 2.6. Statistical Analysis

The analysis was performed using SPSS Version 21 [19], starting with a normality test using the Shapiro–Wilk method. When the *p* value was > 0.05, the data were considered normally distributed, and the analysis continued with a one‐way analysis of variance (ANOVA) test. On the other hand, when the *p* value was < 0.05, the data were considered non‐normally distributed, and analysis continued with the Kruskal–Wallis test. When the results of the one‐way ANOVA or Kruskal–Wallis test showed significant differences between treatments, a post hoc test was performed. Post hoc tests were necessary to identify subgroups that showed significant differences. Correlation and linear regression tests were also conducted to determine the relationship between pineapple waste storage time and total protein content and protease activity of extracted bromelain.

## 3. Results and Discussion

### 3.1. Waste Preparation

The use of pineapple crown and peel waste in this study demonstrates the application of the circular economy concept, where waste with no initial economic value is transformed into a valuable raw material [20]. Using pineapple crown and peel waste not only reduces organic waste but also supports sustainability through resource reuse. This study used ripe pineapples that were 8 months old in line with the demand of the processing industry [21]. The fruit was cleaned and then weighed, which ranged from 1.02 to 2.35 kg, with crown and peel weights of 0.21–0.32 kg and 0.13–0.48 kg, respectively. The yield was 9.73 kg of crown and 11.540 kg of peel from 70.93 kg of pineapple fruit. The percentages of crown and peel weight to the total weight of pineapple fruit were approximately 14%–18% and 15%–21%, respectively. These values are in the range of the crown and peel weight percentage, namely 10%–25% [22] and 10%–30% [21], respectively.

Pineapple crown waste was stored at 29 ± 1°C and a humidity of 70 ± 2%, showing that the longer the storage period, the drier the crown. On the 7th day, the samples started to rot and became infested with microbes. Meanwhile, peels were stored at 4 ± 0.5°C and a humidity of 40 ± 2% due to the high moisture content. Peel waste was stored in a refrigerator, stacked and uncovered, to mimic cold storage conditions. During storage, peel waste dried out and started to show signs of spoilage on the 12th day. This spoilage occurs because stacked storage in containers creates humidity, which supports the growth of microorganisms [23]. After waste preparation, the moisture content of crowns and peels was determined. This step is important because pineapple fruits contain 85% moisture content [24], which affects the decomposition and stability of bromelain in pineapples.

### 3.2. Bromelain Yield

Moisture content on Day 0 for pineapple crown and peel waste was 11.56% and 85.79%, respectively. These values are similar to other studies, which reported values of 18.7% for crowns [25] and 86% for peels [26]. Moisture content continued to decrease daily due to evaporation of water and volatile compounds [27]. Pineapple peels are known to contain ethyl acetate and isopentyl acetate (esters) as the main volatile compounds [28]. During storage, heat and mass transfer occur from the waste to the environment. Heat is transferred to the surface through diffusion and is released into the environment through convection. The heat release occurs simultaneously with the evaporation of water and volatile compounds, causing pineapple peels to lose mass and shrink [29].

Crown and peel waste was extracted using distilled water, as bromelain has a solubility of 1 mg/mL in distilled water [30]. The extract was filtered, and 96% ethanol was added to the filtrate to precipitate bromelain [31, 32]. Ethanol causes denaturation because it disrupts the hydrophobic interactions in the globular protein core. Denaturation causes protein to break down, exposing the hydrophobic regions in the globular protein core to water molecules, leading to precipitation [33, 34]. The precipitation process facilitates the separation of bromelain [35]. The filtrate near the precipitate was centrifuged at 4°C to maintain enzyme stability [36]. The entire precipitate was dried at 30°C to remove ethanol, because it can be evaporated at low temperatures [37]. Denaturation by organic solvents, such as ethanol, is reversible; hence, ethanol evaporation causes bromelain renaturation [32, 33]. The dried powder represents bromelain, which is light brown for the crown and brownish‐white for the peel. Moisture content and percent yield are shown in Table 1.

**TABLE 1:** Moisture content and percentage yield of bromelain from pineapple waste.

Pineapple crown			Pineapple peel		
Days	Moisture content (%)	Yield (%)	Days	Moisture content (%)	Yield (%)
0	17.56	0.154 ± 0.012	0	85.790	0.081 ± 0.007
1	16.24	0.175 ± 0.016	4	79.520	0.082 ± 0.008
3	15.42	0.182 ± 0.009	8	70.890	0.086 ± 0.011
5	12.19	0.108 ± 0.007	12	60.320	0.083 ± 0.005
7	10.83	0.157 ± 0.011	16	49.740	0.080 ± 0.017
			20	40.570	0.083 ± 0.006

After extraction, total protein content was determined. This is important because the simple extraction method followed by ethanol precipitation can precipitate proteins other than bromelain and also precipitate carbohydrates, which are abundant in pineapple crown and peel waste. Ethanol, as an antisolvent precipitation, reduces the solubility of protein and polysaccharides in aqueous plant extracts, resulting in mixed precipitations rather than purified enzymes. Ethanol precipitation for protease extracts from *Bromelia* spp. yielded poor purity due to the linkage of bromelain with carbohydrates [38].

### 3.3. Determination of Total Protein Content

The ethanol precipitation method causes bromelain to precipitate along with other compounds that are insoluble in ethanol [32]. Therefore, it is necessary to determine total protein content using the Bradford method, which uses the principle of dye‐binding colorimetry [17]. The Bradford method is based on the interaction between Bradford reagent, Coomassie Brilliant Blue G‐250 (CBB G‐250), and hydrophobic or basic amino acids. This complex causes a shift in the maximum absorbance of CBB G‐250 from 465 to 595 nm, due to a color change from red to blue [39]. BSA consists of 538 amino acid residues [40] and is relatively stable, making it suitable for use as a standard to determine the total protein content [41]. The equation of the BSA standard curve was *y* = 0.0012*x* + 0.2644, with a coefficient of determination of 0.9915, indicating linearity between absorbance and standard concentration [42]. The total protein content in bromelain is shown in Table 2.

**TABLE 2:** Total protein content of bromelain.

Pineapple waste	Total protein content (μg/mL) (*n* = 3)	Percentage of total protein content to bromelain (% w/w)
Crown, days		
0	168.83 ± 3.818	32.467 ± 0.734
1	176.05 ± 7.561	33.856 ± 1.454
3	169.94 ± 8.221	32.681 ± 1.581
5	193.55 ± 1.924	36.519 ± 0.363
7	169.94 ± 2.678	32.064 ± 0.505
Peel, days		
0	149.944 ± 5.092	29.616 ± 1.565^abcdef^
4	188.833 ± 3.819	37.039 ± 1.189^abef^
8	178.833 ± 4.640	35.305 ± 1.259^acdef^
12	199.944 ± 11.097	39.196 ± 1.736^acdf^
16	207.444 ± 4.276	41.222 ± 1.234^abc^
20	223.833 ± 3.333	43.610 ± 0.532^abcdf^
Standard	289.389 ± 4.739	56.743 ± 0.929

*Note:* Storage time did not affect the total protein content in pineapple crown waste (*p* > 0.05), but had a significant effect on pineapple peel (*p* < 0.05).

The total protein content in bromelain was 32.0%–36.5% for the crown and 29.62%–43.61% for the peel. This indicates that nonprotein compounds are soluble in distilled water and precipitate when ethanol is added [32]. According to Nadzirah et al. [43], bromelain is a combination of different thiol endopeptidases and other components, such as cellulase, glucosidase, peroxidase, phosphatase, glycoprotein, and carbohydrates, along with several protease inhibitors. The total protein content was lower than that of standard bromelain (56.993%) and the previous study (45.301%) [32]. This difference occurs due to the age difference between pineapples used, namely 5 months in 2023 and 8 months in 2025. Ripe pineapples have a lower protein content than unripe ones [32].

The total protein content of bromelain from pineapple waste stored for various storage periods tends to increase (Table 2). This increased total protein content may be due to water hydrolyzing protein into smaller peptides, which are more soluble in water, thereby increasing the amount of extracted protein [43, 44]. On the third day for the crown and the eighth day for the peel, total protein content decreased. Further hydrolysis breaks down protein into amino acids or short‐chain peptides, which are soluble but do not precipitate [45]. Total protein levels continued to increase after the 3rd day for crowns and 8 days for peels, along with a decrease in the moisture content of the waste. The decreased moisture content indicated the evaporation of water and volatile compounds from the waste, leading to the aggregated amino acids and peptides, which allow reprecipitation and increase the total protein content [46]. The highest total protein content of bromelain in crowns and peels was 193.55 ± 1.924 and 223.833 μg/mL, respectively.

A normality test using the Shapiro–Wilk method on the total protein content of crown bromelain showed that the data were not normally distributed. Therefore, analysis using the Kruskal–Wallis method showed no significant difference (*p* < 0.05). A Spearman correlation test [47] further showed no relationship between storage time and total protein content (*p* > 0.05). A linear regression test showed that the storage time of pineapple crown waste did not affect total protein content (*p* > 0.05).

A normality test conducted using the Shapiro–Wilk method on the total protein content of peel bromelain showed that the data were normally distributed (*p* > 0.05), hence, one‐way ANOVA indicated a significant difference (*p* < 0.05). The Bonferroni post hoc test showed a significant difference (*p* < 0.05) as depicted in Table 2. Furthermore, the Pearson correlation test [47] demonstrated a strong correlation with a positive direction between storage time of peel waste and total protein content (*p* < 0.05) with a correlation value of 0.914. This means that the longer the storage time of peel waste, the higher the total protein content. The linear regression test produced a linear equation of *y* = 159.587 + 3.188*x*, where *y* is the total protein content and *x* is the storage time (days). The regression coefficient value of 3.188 indicates that each additional day of storage time will increase the total protein content by 3.188 units. The results showed that the storage time of peel waste had an 83.5% effect on the variation in the total protein content of bromelain (*p* < 0.05; *R* ^2^ = 0.835).

Next, a protease activity test was performed to determine the conformational compatibility of bromelain with casein as its substrate. This is crucial because during the extraction process, bromelain’s conformation can change, resulting in decreased or even complete loss of protease activity. Bromelain relies on native conformation for catalytic activity, and structural dynamics around the active site are critical determinants of proteolytic function. Changes in conformation caused by extraction treatments, solvent interactions, or physicochemical disturbances can lead to reduced or complete loss of enzyme activity [48].

### 3.4. Determination of Protease Activity

Bromelain protease activity was determined by measuring the amount of tyrosine formed from the casein degradation [49]. A standard curve was constructed with the equation of *y* = 0.0004*x* + 3.1001. The coefficient of determination of 0.9915 indicated a linear relationship between absorbance and the standard tyrosine concentration [41]. Bromelain protease activity is shown in Table 3.

**TABLE 3:** Protease activity of bromelain.

Waste	Tyrosine concentration (μg/mL) (*n* = 3)	Protease activity (IU/mg)
Crown, days		
0	275.583 ± 47.258	37.049 ± 6.353a
1	278.916 ± 32.627	37.497 ± 4.386a
3	299.75 ± 17.500	40.298 ± 2.352a
5	315.583 ± 21.262	41.626 ± 2.804
7	351.416 ± 5.294	46.352 ± 0.686a
Peel, days		
0	222.250 ± 6.614	28.152 ± 0.838
4	213.917 ± 12.583	27.096 ± 1.594
8	212.250 ± 2.500	26.885 ± 0.317
12	227.250 ± 6.614	28.785 ± 0.838
16	234.750 ± 13.919	29.735 ± 1.763
20	229.750 ± 15.612	29.102 ± 1.978
Standard	183.917 ± 1.443	23.296 ± 0.183

*Note:* Storage time did not affect protease activity of pineapple peel waste (*p* > 0.05), but had a significant effect on pineapple crown waste (*p* < 0.05).

Protease activity of bromelain from crown and peel waste stored for various storage times ranged from 37.049 to 46.352 IU/mg and 26.885–29.735 IU/mL, respectively (Table 3). This activity was higher than standard bromelain (27.226 IU/mL), but lower than the previous study (46.775 ± 0.159 IU/mg) [32]. These variations were due to differences in pineapple ripeness. The previous study used 5‐month‐old pineapple, while this study used 8‐month‐old pineapple. At a certain level of ripeness, bromelain protease activity decreases [50].

The activity of crown bromelain increased from Day 0 to 7 and reached the highest value on Day 7 at 46.352 ± 0.686 IU/mg. Peel bromelain decreased until Day 8, then increased and reached the highest value on Day 16 at 29,735 IU/mg, before decreasing again on Day 20. Enzymes require a certain amount of water in the structure to maintain natural conformation [51]. Moisture content of crown and peel decreases over time, which causes changes in the enzyme structure, including partial alterations in the tertiary structure, known as partial denaturation. During the partial denaturation phase, changes in enzyme conformation occur, resulting in decreased enzyme activity, as evidenced by the reduction in protease activity of peel bromelain from Day 0 to Day 8 [52]. However, changes in enzyme structure to a certain extent can increase catalytic activity. This is because the enzyme structure becomes more open and the active site is more exposed. The more exposed active site of the enzyme can be observed from the increase in protease activity from Day 0 to 7 for crown bromelain and from Day 8 to 16 for peel bromelain. Continuous changes in the enzyme structure cause alteration in the active site, resulting in decreased activity [53]. Decreased protease activity of peel bromelain by 0.633 IU/mg was observed on Day 20 compared to Day 16. The waste storage time was stopped on Day 20 because protease activity had decreased. Pineapple peel waste provided optimal bromelain enzyme activity until Day 16.

The Shapiro–Wilk normality test for crown and peel bromelain [47] showed a normal distribution (*p* > 0.05), followed by one‐way ANOVA, which indicated a significant difference (*p* < 0.05) for crown bromelain, but no significant difference (*p* > 0.05) for peel bromelain. The Bonferroni post hoc test for crown bromelain showed a significant difference between protease activity after 7 days of storage and for 0, 1, and 3 days (Table 3). The Pearson correlation test for crown bromelain demonstrated a positive relationship between storage time of crown waste and protease activity (*p* < 0.05). A linear regression test showed that the storage time of pineapple crown waste affected protease activity of the crude bromelain enzyme (*p* < 0.05). Storage time affected protease activity by 49%, while the remainder was influenced by other factors (*R* ^2^ = 0.49). The Pearson correlation test for peel bromelain showed a relationship between storage time of peel waste and protease activity, with a correlation value of 0.469 (moderate correlation with a positive direction). This implies that the length of storage of peel waste will be followed by an increase in protease activity. Subsequently, a linear regression test was carried out, which produced a linear equation *y* = 27.252 + 0.104*x*, where *y* is protease activity and *x* is storage time (days). The regression coefficient value of 0.104 indicates that each additional day of storage time will increase protease activity value by 0.104 units. The test results showed that pineapple peel storage time had a 22% effect on the variation in protease activity (*p* = 0.05; *R* ^2^ = 0.220).

The results of this study support the use of pineapple crown and peel waste as a source of bromelain. The stability of bromelain can be maintained from pineapple crown and peel waste stored under appropriate conditions, namely 7 days at 29 ± 1°C and humidity of 70 ± 2% for crown and 20 days at 4 ± 0.5°C and humidity of 40 ± 2% for peel. This is important to ensure the availability of bromelain as a raw material. Our previous studies have shown that bromelain has immunomodulatory activity [54] and can be formulated into granules for dietary supplements [55].

## 4. Conclusion

In conclusion, pineapple crown and peel waste can be stored for 7 days at 29 ± 1°C and humidity of 70 ± 2% and 20 days at 4 ± 0.5°C and humidity of 40 ± 2%, respectively. Total protein content and protease activity were 169.94 ± 2.68 μg/mL and 46.35 ± 0.69 IU/mg for crown bromelain and 229.75 ± 15.61 μg/mL and 29.10 ± 1.98 IU/mg for peel bromelain. These results suggest that pineapple crown waste has more potential than peel as a source of bromelain.

## Author Contributions

Conceptualization, Nyi Mekar Saptarini; methodology, Driyanti Rahayu and Danni Ramdhani; formal analysis, Virgiana Keisha Rajabi Johni Sudirman and Delphine Wirawan; investigation, Driyanti Rahayu and Danni Ramdhani; resources, Nyi Mekar Saptarini; data curation, Driyanti Rahayu; writing–original draft preparation, Virgiana Keisha Rajabi Johni Sudirman and Delphine Wirawan; writing–review and editing, Nyi Mekar Saptarini and Danni Ramdhani; supervision, Driyanti Rahayu; project administration, Nyi Mekar Saptarini.

## Funding

This​ study received university internal funding no. 2104/UN6.0/TU.00/2025, and the APC was funded by the Directorate of Research and Community Service and Innovation Universitas Padjadjaran.

## Disclosure

The authors have read and agreed to the published version of the manuscript.

## Conflicts of Interest

The authors declare no conflicts of interest.

## Data Availability

The data that support the findings of this study are available from the corresponding author upon reasonable request.
